# Quantitative [^99m^Tc]Tc-MDP SPECT/CT correlated with [^18^F]NaF PET/CT for bone metastases in patients with prostate cancer

**DOI:** 10.1186/s40658-022-00513-8

**Published:** 2022-12-05

**Authors:** Kenichi Tanaka, Takashi Norikane, Katsuya Mitamura, Yuka Yamamoto, Yukito Maeda, Kengo Fujimoto, Yasukage Takami, Mariko Ishimura, Hanae Arai-Okuda, Yoichiro Tohi, Nobuyuki Kudomi, Mikio Sugimoto, Yoshihiro Nishiyama

**Affiliations:** 1 Department of Radiology, Faculty of Medicine, Kagawa University, 1750-1 Ikenobe, Miki-cho, Kita-gun, Kagawa 761-0793 Japan; 2 Department of Clinical Radiology, Kagawa University Hospital, Miki-cho, Kagawa Japan; 3 Department of Urology, Faculty of Medicine, Kagawa University, Miki-cho, Kagawa Japan; 4 Department of Medical Physics, Faculty of Medicine, Kagawa University, Miki-cho, Kagawa Japan

**Keywords:** SPECT, PET, SUV, Bone metastases, Prostate cancer

## Abstract

**Background:**

The purpose of the present study was to elucidate the correlation between standardized uptake value (SUV) and volume-based parameters measured by quantitative [^99m^Tc]Tc-methylene diphosphonate (MDP) single photon emission computed tomography (SPECT)/CT and [^18^F]-sodium fluoride ([^18^F]NaF) positron emission tomography (PET)/CT in the assessment of bone metastases in patients with prostate cancer.

**Methods:**

The study included 26 male prostate cancer patients with confirmed or suspected bone metastases who underwent both [^99m^Tc]Tc-MDP SPECT/CT and [^18^F]NaF PET/CT studies. Skeletal lesions visible on both SPECT/CT and PET/CT were classified as benign or metastases. The maximum SUV (SUVmax), peak SUV (SUVpeak), mean SUV (SUVmean), metabolic bone volume (MBV), and total bone uptake (TBU) were calculated for every lesion showing abnormal uptake.

**Results:**

A total of 202 skeletal lesions (147 benign and 55 metastases) were detected in the 26 patients. Strong significant correlations were noted between SPECT/CT and PET/CT for the SUV- and volume-based parameters (all *P* < 0.001). The SUVmax, SUVpeak, SUVmean, and TBU values obtained with SPECT/CT were significantly lower than the corresponding values obtained with PET/CT (all *P* < 0.001). The MBV in SPECT/CT was significantly higher than that in PET/CT (*P* < 0.001). All SUV- and volume-based parameters obtained with both SPECT/CT and PET/CT for metastatic lesions were significantly higher than the corresponding parameters for benign lesions (*P* values from 0.036 to < 0.001).

**Conclusions:**

These preliminary results demonstrate that the SUV- and volume-based parameters for bone uptake obtained with quantitative SPECT/CT and PET/CT are strongly correlated in patients with prostate cancer. The SUV parameters obtained with SPECT/CT were significantly lower than those obtained with PET/CT, whereas the uptake volume obtained with SPECT/CT was significantly higher than that obtained with PET/CT.

## Background

In prostate cancer, the most common malignancy in men, bone scintigraphy is mainly used for assessment of high-risk patients, in whom the extent of bone metastases has been reported to be an independent prognostic factor [1, 2]. Single photon emission computed tomography (SPECT) with ^99m^Tc-labeled diphosphonates, such as [^99m^Tc]Tc-methylene diphosphonate (MDP) is used to assess bone metastases from prostate cancer. Although positron emission tomography (PET) with [^18^F]-sodium fluoride ([^18^F]NaF) is a more sensitive method than SPECT with ^99m^Tc-labeled diphosphonates for detecting bone metastases [3, 4], it is not widely used because of high cost and limited availability.

SPECT has traditionally been used in a non-quantitative manner; namely, images are interpreted using relative intensity values instead of absolute values of radiotracer concentration [5]. However, in recent years, the wide acceptance of integrated SPECT/CT scanners and the development of iterative reconstruction algorithms have enabled the clinical use of quantitative SPECT as well as PET [6]. The most commonly used semiquantitative PET parameter is the standardized uptake value (SUV). Owing to its simplicity, the maximum SUV (SUVmax) inside a lesion is mainly used to clinically represent the intensity of radioactivity within the lesion. Nowadays, volume-based parameters such as metabolic tumor volume and total lesion glycolysis have also proven to be helpful for assessments.

[^18^F]NaF PET/CT has very good accuracy for the diagnosis of bone metastases from prostate cancer [3, 4, 7]. However, it is not a routine modality. Moreover, although recent developments in SPECT/CT technology enable it to be used to calculate the SUV, it has not been fully compared with PET/CT as the gold standard. Thus, there is a need to standardize the evaluation and interpretation of SPECT/CT and PET/CT findings, especially in the assessment of therapeutic response. Only a single study has previously reported a direct comparison between bone SPECT/CT and [^18^F]NaF PET/CT using SUV-based parameters, not volume-based parameters, for measuring metastatic bone uptake [4]. This prompted us to investigate the correlation between SUV- and volume-based parameters measured by quantitative [^99m^Tc]Tc-MDP SPECT/CT and [^18^F]NaF PET/CT to assess bone metastases in patients with prostate cancer.

## Methods

### Patients

We conducted a retrospective analysis of prospectively collected data. The prospective study consisted of 48 consecutive prostate cancer patients with confirmed or suspected bone metastases who underwent [^18^F]NaF PET/CT between November 2018 and May 2021. All patients provided written informed consent, and the study protocol was approved by our institutional ethical review committee. Among these patients, 26 male patients (mean age, 72.0 years; age range, 56–89 years) were selected for this retrospective analysis. Eligible patients had undergone both [^99m^Tc]Tc-MDP SPECT/CT and [^18^F]NaF PET/CT studies within 2 months. Fifteen patients were newly diagnosed with high-risk prostate cancer, and the remaining 11 were suspected of showing recurrence or progression later in the course of the disease. The median interval between the [^99m^Tc]Tc-MDP SPECT/CT and [^18^F]NaF PET/CT scans was 8 days (range, 1–50 days). This retrospective data collection was compliant with our institutional ethical review committee, which waived the requirement for informed consent.

### [^99m^Tc]Tc-MDP SPECT/CT imaging

At 210 min after intravenous injection of [^99m^Tc]Tc-MDP (740 MBq), SPECT scans of the entire axial skeleton were obtained using two Symbia T16 SPECT/CT scanners (Siemens Healthcare, Erlangen, Germany) with the following parameters: low-energy high-resolution collimators, 180 projections over 360° with 8 s/ step, 128 × 128 matrix, 4.8 × 4.8 mm pixel size, and 4.8 mm slice thickness. Unenhanced low-dose CT data from the same area were used for attenuation correction and image fusion. SPECT data were reconstructed using a three-dimensional (3D) ordered-subset expectation–maximization (OSEM) algorithm with 15 iterations, six subsets incorporating correction with resolution recovery, scatter correction, and an 11-mm Gaussian filter.

### [^18^F]NaF PET/CT imaging

All acquisitions were performed using a Biograph mCT 64-slice PET/CT scanner (Siemens Healthcare; Erlangen, Germany). Emission data were obtained 90 min after intravenous injection of [^18^F]NaF (5 MBq/kg) from the knee to the skull (2 min per bed position). Unenhanced low-dose CT data from the same area were used for attenuation correction and image fusion. PET data were reconstructed in a 256 × 256 matrix with a pixel size of 3.18 × 3.18 mm and slice thickness of 5.0 mm using a fully 3D OSEM algorithm with two iterations, 21 subsets incorporating correction with a point-spread function and time-of-flight model, scatter correction, and a 5-mm Gaussian filter.

### Image analysis

Skeletal lesions visible on both SPECT/CT and PET/CT examinations were interpreted. On the basis of the corresponding morphologic findings on the CT images of SPECT/CT and PET/CT [8], skeletal lesions with abnormal uptake were classified as benign or metastases by the consensus of a board-certified nuclear medicine physician and a board-certified radiologist. The patients’ medical records were reviewed to obtain the final diagnosis of equivocal lesions.

The SUV- and volume-based analyses using SPECT/CT and PET/CT were performed by a board-certified nuclear medicine physician using GI-BONE, a commercially available software package (AZE, Tokyo, Japan), and Syngo.via (Siemens Healthcare, Erlangen, Germany), respectively. The volume of interest (VOI) of the contour of the identified skeletal lesion was placed using 3D reconstructed images. A threshold was selected by visual inspection to best fit the contour of the abnormal uptake. The SUVmax, peak SUV (SUVpeak), defined as the mean concentration of activity within a 1-cm^3^ spherical VOI centered on the highest voxel within the lesion, mean SUV (SUVmean), metabolic bone volume (MBV), defined as lesion volume with uptake, and total bone uptake (TBU), calculated as SUVmean × MBV, were calculated for every lesion showing abnormal uptake.

### Statistical analysis

Data were analyzed using SPSS statistical software (version 28; IBM). Spearman’s rank correlation coefficients were used to determine the degree of correlation between SPECT and PET parameters. The Wilcoxon signed-rank test was used to compare the differences between SPECT and PET parameters. Differences between benign and metastatic lesions were analyzed using the Mann–Whitney *U*-test. Differences were considered statistically significant at *P* values less than 0.05.

## Results

**Fig. 1:**
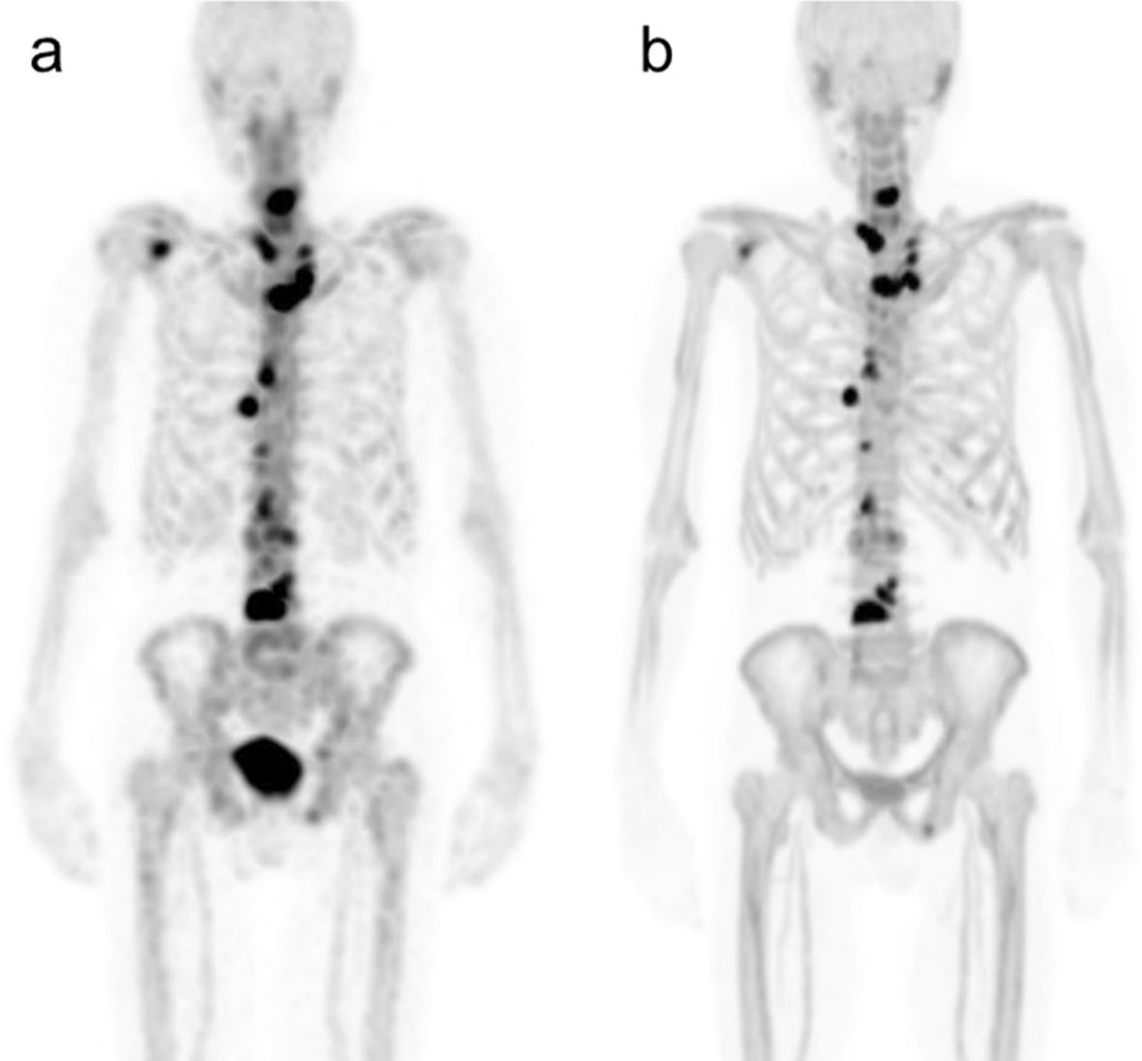
[^99m^Tc]Tc-MDP SPECT (**a**) and [^18^F]NaF PET (**b**) maximum intensity projection images of an 89-year-old male with prostate cancer showing multiple bone metastases

A total of 202 skeletal lesions, including 147 benign and 55 metastases, were detected in 26 patients. Table 1 shows the CT findings of lesions identified as benign on SPECT/CT or PET/CT images with abnormal uptake. Typical SPECT and PET images are shown in Fig. 1. The median SUV threshold used for segmentation was 60% (range, 10%-80%) of the SUVmax in SPECT and 40% (range, 10%-70%) of the SUVmax in PET.

**Table 1:** Location and CT findings of 147 benign skeletal lesions identified using SPECT/CT or PET/CT

Location	Findings (*n*)
Facial bones	Sinusitis (6), dental disease (12)
Cervical spine	Osteophytes (12), osteoarthritis (12)
Thoracic spine	Osteophytes (23), osteoarthritis (1)
Lumbar spine	Osteophytes (22), osteoarthritis (12), fracture (5)
Pelvic bones	Osteoarthritis (3), avulsion injury (1)
Thoracic cage	Arthritic changes at the acromioclavicular (19) and sternoclavicular (1) joints, fracture (14), postoperative change (1)
Long bones	Subchondral cyst (3)

**Fig. 2:**
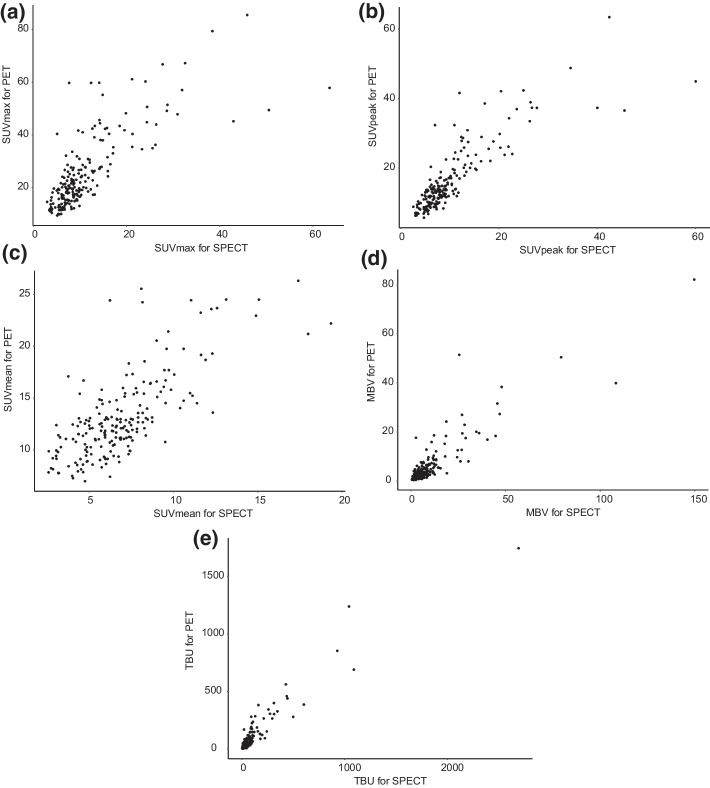
Scatter plots showing the correlation between SPECT and PET parameters in SUVmax (**a**) (*ρ* = 0.764, *P* < 0.001), SUVpeak (**b**) (*ρ* = 0.838, *P* < 0.001), SUVmean (**c**) (*ρ* = 0.679, *P* < 0.001), MBV (**d**) (*ρ* = 0.807, *P* < 0.001), and TBU (**e**) (*ρ* = 0.874, *P* < 0.001)

There were strong and statistically significant correlations between SPECT/CT and PET/CT parameters in SUVmax (Fig. 2a, *ρ* = 0.764, *P* < 0.001), SUVpeak (Fig. 2b, *ρ* = 0.838, *P* < 0.001), SUVmean (Fig. 2c, *ρ* = 0.679, *P* < 0.001), MBV (Fig. 2d, *ρ* = 0.807, *P* < 0.001), and TBU (Fig. 2e, *ρ* = 0.874, *P* < 0.001).

Table 2 shows the SPECT/CT and PET/CT parameters for all skeletal lesions. The SUVmax, SUVpeak, SUVmean, and TBU were all significantly lower in SPECT/CT than in PET/CT (all *P* < 0.001). In contrast, the MBV in SPECT/CT was significantly higher than that in PET/CT (*P* < 0.001).

**Table 2:** Relationship between SPECT and PET parameters for all skeletal lesions in patients with prostate cancer

	SPECT	PET	*P* value
SUVmax	8.66 (6.69–12.67)	20.50 (15.87–29.76)	< 0.001
SUVpeak	7.85 (5.88–10.99)	13.25 (10.05–18.85)	< 0.001
SUVmean	6.55 (5.01–8.04)	12.31 (10.41–14.82)	< 0.001
MBV	5.24 (2.65–9.98)	3.02 (1.66–6.10)	< 0.001
TBU	34.20 (13.95–75.27)	36.00 (17.71–85.82)	< 0.001

Data are presented in terms of the median (IQR) values

*SUV* Standardized uptake value, *SUVmax* Maximum SUV, *SUVpeak* Peak SUV, *SUVmean* Mean SUV, *MBV* Metabolic bone volume, *TBU* Total bone uptake

Table 3 shows the results of SPECT/CT and PET/CT parameters for benign and metastatic lesions. For SPECT/CT, the SUVmax (*P* < 0.001), SUVpeak (*P* < 0.001), SUVmean (*P* = 0.005), MBV (*P* = 0.002), and TBU (*P* = 0.002) of metastatic lesions were significantly higher than those of benign lesions. For PET/CT, the SUVmax (*P* < 0.001), SUVpeak (*P* < 0.001), SUVmean (*P* < 0.001), MBV (*P* = 0.036), and TBU (*P* = 0.002) of metastatic lesions were also significantly higher than those of benign lesions.

**Table 3:** Relationship between benign and metastatic lesions in terms of SPECT and PET parameters in patients with prostate cancer

	SPECT			PET		
	Benign	Metastatic	*P* value	Benign	Metastatic	*P* value
SUVmax	8.49 (6.45–10.45)	13.08 (7.25–24.40)	< 0.001	19.04 (15.28–24.73)	32.99 (20.30–45.16)	< 0.001
SUVpeak	7.58 (5.65–9.28)	11.68 (6.23–20.29)	< 0.001	12.25 (9.68–15.14)	20.36 (13.60–30.90)	< 0.001
SUVmean	6.35 (4.91–7.48)	7.58 (5.06–10.99)	0.005	11.74 (9.92–13.03)	15.42 (12.45–19.76)	< 0.001
MBV	4.62 (2.41–8.22)	8.48 (3.11–15.66)	0.002	2.63 (1.52–5.12)	3.95 (1.87–10.02)	0.036
TBU	28.25 (13.07–63.06)	52.23 (17.47–176.62)	0.002	31.23 (16.94–66.51)	48.18 (24.58–187.31)	0.002

Data are presented in terms of the median (IQR) values

*SUV* Standardized uptake value, *SUVmax* Maximum SUV, *SUVpeak* Peak SUV, *SUVmean* Mean SUV, *MBV* Metabolic bone volume, *TBU* Total bone uptake

## Discussion

This study assessed the correlations of SUV- and volume-based parameters obtained using quantitative [^99m^Tc]Tc-MDP SPECT/CT and [^18^F]NaF PET/CT in the evaluation of bone metastases in patients with prostate cancer. The findings indicated that SUV- and volume-based parameters obtained using SPECT/CT and PET/CT were strongly correlated.

Even-Sapir et al. qualitatively compared the detection of bone metastases using [^99m^Tc]Tc-MDP SPECT and [^18^F]NaF PET in 44 patients with high-risk prostate cancer and showed that [^18^F]NaF PET was more sensitive than [^99m^Tc]Tc-MDP SPECT [3]. PET shows high sensitivity and resolution, allowing highly accurate whole-body screening of metastases [9]; however, it is expensive and not widely used as a clinical imaging technique for this purpose. In contrast, high-quality SPECT algorithms have recently enabled the clinical use of quantitative SPECT assessments. This prompted us to perform a quantitative analysis to compare SPECT and PET.

Only one direct comparative study has evaluated the correlations between SUV measurements obtained with bone SPECT/CT and [^18^F]NaF PET/CT for metastatic and benign bone uptake [4]. The authors of that study evaluated the correlations between parameters obtained using [^99m^Tc]Tc-hydroxyethylene (HDP) SPECT/CT and [^18^F]NaF PET/CT in bone metastases from breast and prostate cancer and demonstrated strong correlations of SUV parameters between SPECT and PET [4], consistent with the results of the present study. In the present study, the volume-based parameters of the two methods were also strongly correlated. To the best of our knowledge, this is the first direct comparative study to report volume-based parameters using bone SPECT/CT and [^18^F]NaF PET/CT.

Schirrmeister et al. reported that [^18^F]NaF PET was found to be more sensitive and accurate compared with [^99m^Tc]Tc-MDP bone scintigraphy for the detection of osteoblastic and osteolytic metastases in patients with breast cancer [10]. To our knowledge, there are no studies quantitatively comparing [^18^F]NaF PET/CT and bone SPECT/CT for osteolytic bone metastases. Yen et al. evaluated the diagnostic usefulness of [^18^F]NaF PET/CT relative to [^99m^Tc]Tc-MDP whole-body planar bone scintigraphy with no CT in detecting metastatic hepatocellular carcinoma bone lesions that are predominantly osteolytic in nature, and showed that [^18^F]NaF PET/CT has significantly better sensitivity and specificity [11]. Araz et al. reported that [^18^F]NaF PET/CT could detect both osteolytic and osteoblastic metastases as well as bone marrow involvement, and that small lesions were better visualized due to the advantages of greater spatial resolution and better image quality when compared with ^99m^Tc-labeled whole-body bone scan [12]. Another study by Cook et al. suggested that semi-quantitative [^18^F]NaF PET data may be useful as imaging biomarkers for monitoring treatment response in bone metastases following ^223^Ra-chloride treatment [13]. Further studies comparing the use of [^18^F]NaF PET/CT and bone SPECT/CT for assessment not only osteoblastic bone metastases, but also osteolytic bone metastases, are required.

In this study, the SUV parameters obtained with SPECT/CT were significantly lower than those obtained with PET/CT, which is consistent with a previous report [4]. A previous report indicated that the differences in SUV between the two methods were larger for smaller lesions. Thus, some differences were caused by the different spatial resolutions of SPECT and PET scanners [4]: because of the lower spatial resolution, underestimation of the SUV attributable to the partial-volume effect is more significant in SPECT than in PET [4, 5]. Blood protein binding interferes with the extraction of ^99m^Tc-labeled diphosphonates but does not affect the extraction of [^18^F]NaF [14]. Thus, the uptake of [^18^F]NaF was approximately twofold higher and its blood clearance was significantly faster than that of ^99m^Tc-labeled diphosphonates used in bone scintigraphy, resulting in an increased bone-to-background ratio [15]. Similarly, in this study, the median values of SUV parameters in PET were approximately twice those in SPECT. In contrast, the MBV, defined as the lesion volume with uptake, of SPECT was significantly higher than that of PET in this study. This may be due to the selection of a threshold by visual inspection to best fit the contour of abnormal uptake. Gamma-camera SPECT systems have poorer spatial resolution than PET systems [16, 17]. Umeda et al. identified a cut-off SUVmax of 7.0 to assess the bone metastatic burden using [^99m^Tc]Tc-MDP SPECT/CT in patients with prostate cancer [18]. Tabotta and co-workers determined that the optimum SUVmax cut-off to define bone metastases in the spine and pelvis on [^99m^Tc]Tc-2,3-dicarboxy propane-1,1-diphosphonate (DPD) SPECT/CT was 19.5 [19]. The volume with uptake depends on the VOI. Thus, the definition of the SUV threshold for volumetric analysis is an important issue, and further research is needed to establish a threshold.

The most important factors for generating quantitative SPECT and PET data are the scanner calibration and the reconstruction algorithms that correct for photon attenuation and scattering within the object [5]. Reliable calculation of SUV parameters also requires an accurate injection dose, injection time, and patient weight [4]. In a study by Arvola et al., the SUV ratios of the lesion were calculated by dividing the lesion SUV by the background bone activity SUV, which can be calculated without scanner calibration or information about the accurate injection dose or patient weight. This is technically easier to achieve than with an SUV [4]. The authors suggested that SPECT SUV ratios could be used to expand the visual analysis of bone SPECT, especially in follow-up studies to decrease inter-observer variability and standardize SPECT results among patients, imaging scanners, and clinical centers [4].

The main limitations of this study are its retrospective, single-center design, small sample size with only prostate cancer patients, and lack of histopathological analysis for bone metastases. In the future, it will be necessary to consider not only prostate cancer but also other cancer types in studies with larger cohorts. Lesions visible only on either SPECT or PET should also be considered in future studies. A threshold for contouring lesion volume was selected by visual inspection to best fit the contour of abnormal uptake. In this method, the lesion boundary may be easily altered by changing the window width/level. However, this method was better than setting a single threshold for contouring small lesions or lesions with a low radiotracer uptake. Some studies have indicated that gradient-based segmentation methods are better than manual- and threshold-contouring methods [20–22]. Since MBV and TBU highly depend on segmentation, i.e., thresholding, further study is required to address the correlation of these volumetric values between SPECT and PET. Defining the threshold for volumetric analysis is an important issue, and further research is needed to establish this step. Because only skeletal lesions visible on both SPECT and PET images were assessed, no data were provided on their relative diagnostic accuracy, such as the differential diagnosis of benign and metastatic bone lesions. In patients with suspected recurrence or progression, the therapeutic circumstances vary. In the future, it may be clinically useful to develop a new method to delineate SPECT based on PET as the gold standard.

## Conclusions

These preliminary results obtained in a limited patient population demonstrate that the SUV- and volume-based parameters are strongly correlated between SPECT/CT and PET/CT for the evaluation of bone metastases in patients with prostate cancer. The SUV parameters in SPECT/CT were significantly lower than those in PET/CT, although the uptake volume in SPECT/CT was significantly higher than that obtained with PET/CT.

## Data Availability

The data that support the findings of this study are available from the corresponding author on reasonable request.

## Abbreviations

3D: Three-dimensional
DPD: 2,3-Dicarboxy propane-1,1-diphosphonate
HDP: Hydroxyethylene
MBV: Metabolic bone volume
MDP: Methylene diphosphonate
NaF: Sodium fluoride
OSEM: Ordered-subset expectation–maximization
PET: Positron emission tomography
SPECT: Single photon emission computed tomography
SUV: Standardized uptake value
SUVmax: Maximum SUV
SUVmean: Mean SUV
SUVpeak: Peak SUV
TBU: Total bone uptake
VOI: Volume of interest
